# *Kluyveromyces marxianus* developing ethanol tolerance during adaptive evolution with significant improvements of multiple pathways

**DOI:** 10.1186/s13068-019-1393-z

**Published:** 2019-03-22

**Authors:** Wenjuan Mo, Mengzhu Wang, Rongrong Zhan, Yao Yu, Yungang He, Hong Lu

**Affiliations:** 1 State Key Laboratory of Genetic Engineering, School of Life Science, Fudan University, Shanghai, 200438 China; 2 Key Laboratory of Medical Epigenetics and Metabolism, Institutes of Biomedical Sciences, Fudan University, Shanghai, 200032 China; 3 Shanghai Engineering Research Center of Industrial Microorganisms, Shanghai, 200438 China

**Keywords:** *Kluyveromyces marxianus*, Ethanol tolerance, Adaptive evolution, RNA-seq analysis, Multiple-stress tolerance

## Abstract

**Background:**

*Kluyveromyces marxianus*, the known fastest-growing eukaryote on the earth, has remarkable thermotolerance and capacity to utilize various agricultural residues to produce low-cost bioethanol, and hence is industrially important to resolve the imminent energy shortage crisis. Currently, the poor ethanol tolerance hinders its operable application in the industry, and it is necessary to improve *K. marxianus*’ ethanol resistance and unravel the underlying systematical mechanisms. However, this has been seldom reported to date.

**Results:**

We carried out a wild-type haploid *K. marxianus* FIM1 in adaptive evolution in 6% (v/v) ethanol. After 100-day evolution, the KM-100d population was obtained; its ethanol tolerance increased up to 10% (v/v). Interestingly, DNA analysis and RNA-seq analysis showed that KM-100d yeasts’ ethanol tolerance improvement was not due to ploidy change or meaningful mutations, but founded on transcriptional reprogramming in a genome-wide range. Even growth in an ethanol-free medium, many genes in KM-100d maintained their up-regulation. Especially, pathways of ethanol consumption, membrane lipid biosynthesis, anti-osmotic pressure, anti-oxidative stress, and protein folding were generally up-regulated in KM-100d to resist ethanol. Notably, enhancement of the secretory pathway may be the new strategy KM-100d developed to anti-osmotic pressure, instead of the traditional glycerol production way in *S. cerevisiae*. Inferred from the transcriptome data, besides ethanol tolerance, KM-100d may also develop the ability to resist osmotic, oxidative, and thermic stresses, and this was further confirmed by the cell viability test. Furthermore, under such environmental stresses, KM-100d greatly improved ethanol production than the original strain. In addition, we found that *K. marxianus* may adopt distinct routes to resist different ethanol concentrations. Trehalose biosynthesis was required for low ethanol, while sterol biosynthesis and the whole secretory pathway were activated for high ethanol.

**Conclusions:**

This study reveals that ethanol-driven laboratory evolution could improve *K. marxianus*’ ethanol tolerance via significant up-regulation of multiple pathways including anti-osmotic, anti-oxidative, and anti-thermic processes, and indeed consequently raised ethanol yield in industrial high-temperature and high-ethanol circumstance. Our findings give genetic clues for further rational optimization of *K. marxianus*’ ethanol production, and also partly confirm the positively correlated relationship between yeast’s ethanol tolerance and production.

**Electronic supplementary material:**

The online version of this article (10.1186/s13068-019-1393-z) contains supplementary material, which is available to authorized users.

## Background

Utilizing yeasts to ferment on renewable biomass to produce bioethanol is a promising trend for new energy development. Nevertheless, in the late stage of fermentation, yeast has to withstand the damage brought by high-concentrated ethanol [1], and the cell membrane is the major site for ethanol attack [2, 3]. Along with the gradually elevated ethanol levels in a medium, e.g., 2–10% (v/v), cell viability descends correspondingly [4]. When ethanol concentration exceeds the maximum tolerable concentration, cell growth is severely inhibited and yeasts die immediately, finally resulting in the decline of ethanol yields [5]. Therefore, yeast’s ethanol endurance ability essentially determines its capacity of ethanol production, and improving ethanol tolerance as an efficient way to elevate ethanol yield has been widely recognized in fundamental researches and industry application [6, 7].

*Kluyveromyces marxianus*, a type of “non-conventional” yeast in *Kluyveromyces* genus of the family Saccharomycetaceae, has recently been in the limelight for economic cellulosic ethanol production. Besides its ability of ethanol fermentation, *K. marxianus* possesses a number of advantages over *S. cerevisiae*, which has been traditionally used in bioethanol production. *K. marxianus* is the fastest-growing yeast, with the maximum growth rate of 0.80 h^−1^ [8], and *S. cerevisiae* is only 0.37 h^−1^ [9]. *K. marxianus* can even grow at 52 °C due to its notable thermotolerance [9], while *S. cerevisiae* has optimum temperatures ranging between 30 and 37 °C [10, 11]. Also, besides glucose, *K. marxianus* is able to utilize a variety of carbon sources, including inulin, xylose, and lactose [12, 13], which cannot be used by *S. cerevisiae* [9]. Furthermore, ethanol generation prefers an anaerobic environment; thus, the capability to grow under full anaerobiosis is also required for a yeast strain to be used in a fuel ethanol-producing process. *K. marxianus*, like *S. cerevisiae*, is a respiro-fermentative yeast [9], and the batch fermentation of *K. marxianus* in a strict anaerobic environment at 37 °C reached ethanol concentrations significantly higher than in aerobiosis [14]. Based on the above, *K. marxianus* could be an ideal yeast for industrial bioethanol production. Currently, *K. marxianus* can only bear the maximum 6% (v/v) ethanol, which is measured by the growth ability in shake flask culture in YPD medium with 6% ethanol at 30 °C [11]. The low ethanol tolerance leads to its low ethanol yield and is the major bottleneck to block its practical industry application so far [15].

Yeast’s ethanol resistance is a multiple-gene-regulated complex phenotype. Currently, in the case of *S. cerevisiae*, there are hundreds of genes involved in ethanol response, covering ethanol metabolism, glycolysis, plasma membrane composition, protein folding, cell wall biogenesis, lipid metabolism, responsive reactor generation, etc. [3, 16–18]. Besides the modern genetics approaches to improving *S. cerevisiae* ethanol tolerance, e.g., global transcription machinery engineering [1, 19, 20], transposon mutation [21], genome shuffling [22, 23], and gene engineering [24], the adaptive experimental evolution is also applied as a traditional and ‘natural’ method to develop its ethanol resistance [25, 26]. Over a 2-year study on *S. cerevisiae* adaptation to increasing levels of ethanol, Voordeckers et al. found that ethanol tolerance increased from 6% to 12% (v/v), which is measured by the ethanol concentrations in turbidostat cultures using Sixfors reactors with YPD medium at 30 °C, and diploid was the stable ploidy for *S. cerevisiae* tolerant to ethanol [25]. Moreover, after adaptive laboratory evolution in acetic acid, *S. cerevisiae* became multiple-stress tolerant, i.e., simultaneously resistant to osmotic, thermic, oxidative, ethanol, and organic acid stresses [27]. This stress-cross-tolerance phenomenon indicates multiple stresses in yeast may share some common anti-stress pathways.

To date, there are only a few reports about *K. marxianus*’ ethanol tolerance [28, 29]. Diniz et al. used lactose as a carbon source, and compared the transcriptomes of stressed and non-stressed *K. marxianus* during short-term 6% (v/v) ethanol exposure, and then found that when faced ethanol, the central metabolic flow, including TCA cycle, was impaired, and unsaturated fatty acid biosynthesis also decreased [28]. Li and colleagues screened a random mutagenesis library of *K. marxianus* TATA-binding protein Spt15, and obtained the best tolerant strain with maximum tolerance to 5% (v/v) ethanol, while the original wild-type strain could only bear 2% (v/v) [29], which is measured by the growth ability in shake flask culture in YPD medium containing the corresponding ethanol concentrations at 30 °C. Similar to *S. cerevisiae*, the ethanol resistance of *K. marxianus* is also a complex process involving multiple genes and various physiological pathways [29], and is hard to be upgraded by rational engineering approach, due to the molecular basis of its ethanol tolerance far from fully understood.

In this study, to develop high-ethanol-tolerant *K. marxianus* strains and unravel genetic determinants of the possible tolerance improvement, laboratory evolution and an RNA-seq analysis were carried out. *K. marxianus* has undergone adaptive evolution driven by 6% (v/v) ethanol, and after 100 days, the evolved KM-100d strains were derived with ethanol tolerance elevated from 7 to 10% (v/v). Although no ploidy change or dominant mutation was detected in the KM-100d population by DNA analysis, the RNA-seq analysis revealed that transcription of KM-100d had been totally reprogrammed in the evolution. Pathways involving in ethanol tolerance, such as protein fold, anti-oxidation, anti-osmotic pressure, membrane lipid biosynthesis, cell wall biogenesis, and secretory pathway, were essentially strengthened to ready for upcoming ethanol stress. Furthermore, as suggested by RNA-seq data, KM-100d may also develop resistance to osmotic, oxidative, and thermic stresses, and was validated by cell viability test. Finally, the improved tolerance of *K. marxianus* indeed led to the increased ethanol production in a multiple-stress environment. These findings provide an evolved *K. marxianus* yeast for industrial bioethanol production, and support theoretical fundament for finding new routes to rationally improve *K. marxianus*’ ethanol tolerance for industrial service.

## Results

### Improved resistance to ethanol after a 100-day evolution

**Fig. 1:**
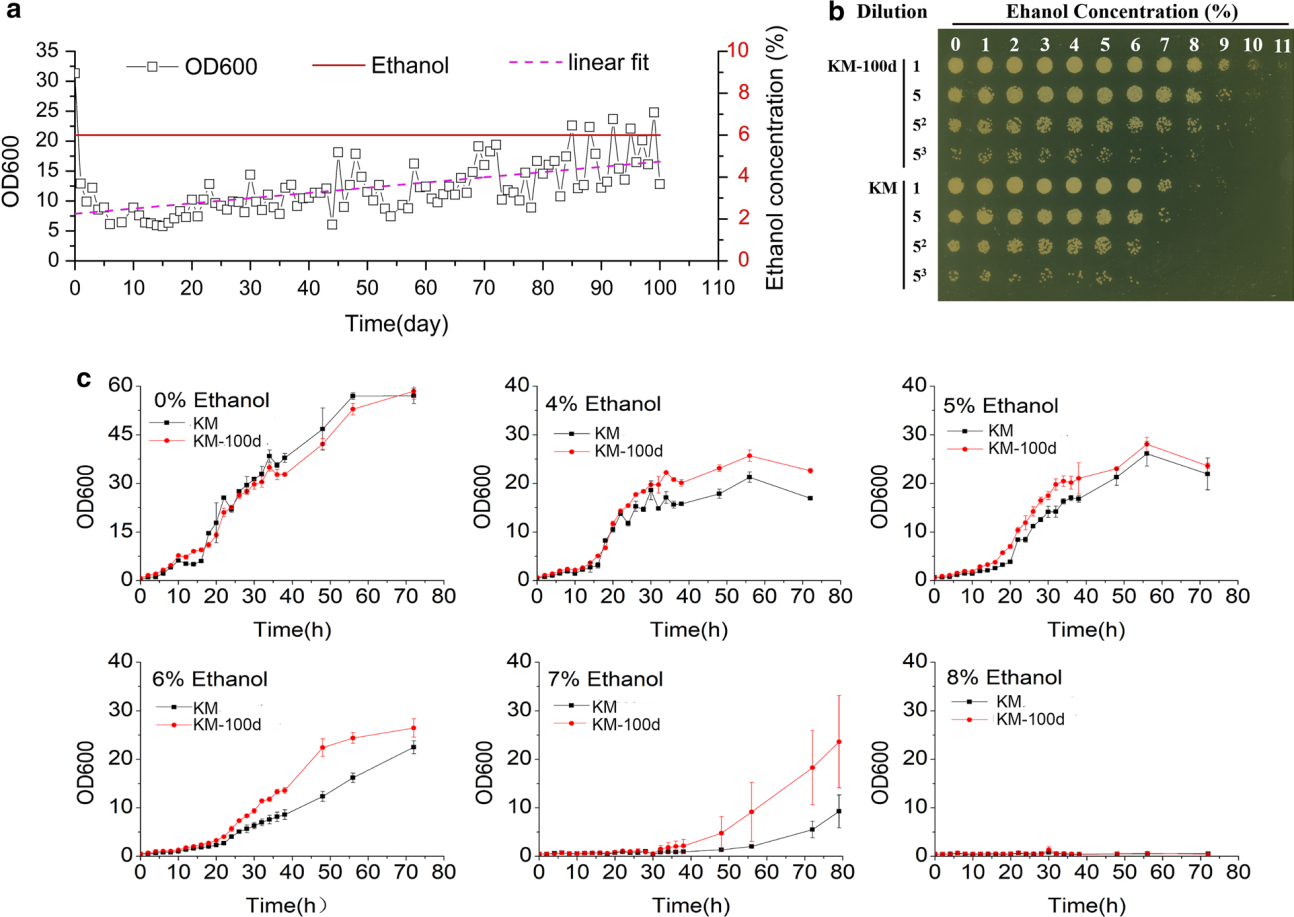
*K. marxianus* evolution in 6% (v/v) ethanol. **a** Daily OD_600_ value of *K. marxianus* population during evolution. Cells were every day transferred and subcultured into a fresh medium containing 6% (v/v) ethanol, with the same initial OD_600_ of 0.6. Then after incubation at 30 °C in an orbital shaker for 24 h, OD_600_ was measured to record cell growth. **b** Spotted dilution analysis for ethanol tolerance between pre- and post-evolution. KM and KM-100d cells were inoculated on liquid medium with ethanol at one of the gradient concentrations: 0, 1, 2,…, 11% (v/v), respectively, and incubated at 30 °C for 3 days. A cell suspension of 10 OD_600_ from the liquid medium was 5-fold serially diluted and spotted onto a YPD plate and cultured at 30 °C for 2 days. **c** Growth profiles of KM and KM-100d at different ethanol concentrations. The red curve is for KM-100d, and the black one is for KM. During growth profile measurement, KM and KM-100d were both carried out in biological triplicate

The wild-type haploid *K. marxianus* strain was cultured in medium with 6% (v/v) ethanol at 30 °C for 100 days about 450 generations (see Methods for details). There was a continuous increase in biomass (measured by OD_600_ daily) during this period (Fig. 1a), indicating cell survival under ethanol stress may be improved. At the end, we obtained a *K. marxianus* population with greatly improved resistance to ethanol (Fig. 1b). Throughout, KM refers to the raw strain before evolution, and KM-100d refers to the *K. marxianus* population after the 100-day evolution. The maximum ethanol resilience for KM was 7% (v/v) and for KM-100d, it was up to 10% (Fig. 1b). We compared growth profiles between KM and KM-100d in culture media with different ethanol levels (0%, 4%, 5%, 6%, 7%, 8% v/v, Fig. 1c). In the absence of ethanol, there was no significant difference between the strains. Their difference increased with the increase of ethanol level and reached the maximum with 6% (v/v) ethanol in culture media. In such circumstance, after 48 h, the biomass of KM-100d was nearly twofold higher than that of KM. Both of the strains showed retarded growth in medium with 7% ethanol and failed to grow in the medium with 8% ethanol. In addition, KM-100d also showed remarkable higher maximum growth rate than KM at 6% ethanol (Additional file 1: Figure S1).

### DNA analysis suggest that *K. marxianus* has no change of ploidy or critical genes during adaptive evolution

To elucidate DNA alterations in KM-100d, we conducted DNA ploidy analysis and DNA mutation identification. During the adaptive evolution, the DNA content of *K. marxianus* population has little change (Additional file 1: Figure S2); thus, no ploidy change took place. By DNA-seq analysis of KM and KM-100d, which were both mapped to the reference genome of *K. marxianus* DMKU 3-1042 [30], the SNP sites between KM and KM-100d were obtained (Additional file 2). Among the identified 57 SNPs, only 4 sites were dominant in KM-100d population. Three of them are located in the coding region of SAN1, YAP1, and KHT2 genes; the other one is at the 445 bp upstream of ERG26. The 1324 site of SAN1 was mutated from C to T, and consequently the corresponding amino acid was transformed from arginine to cysteine. However, according to analysis of the Pfam 32.0 [31], this mutation does not fall into any identified protein domain. Both the mutations in YAP1 and KHT2 are synonymous mutations without protein change. The above findings suggest that changes in DNA context are far from sufficient to support such phenotype improvement in KM-100d, and transcriptional reprogramming must contribute to this. Therefore, we further carried out RNA-seq analysis.

### A global rewiring induced by the 100-day evolution

**Fig. 2:**
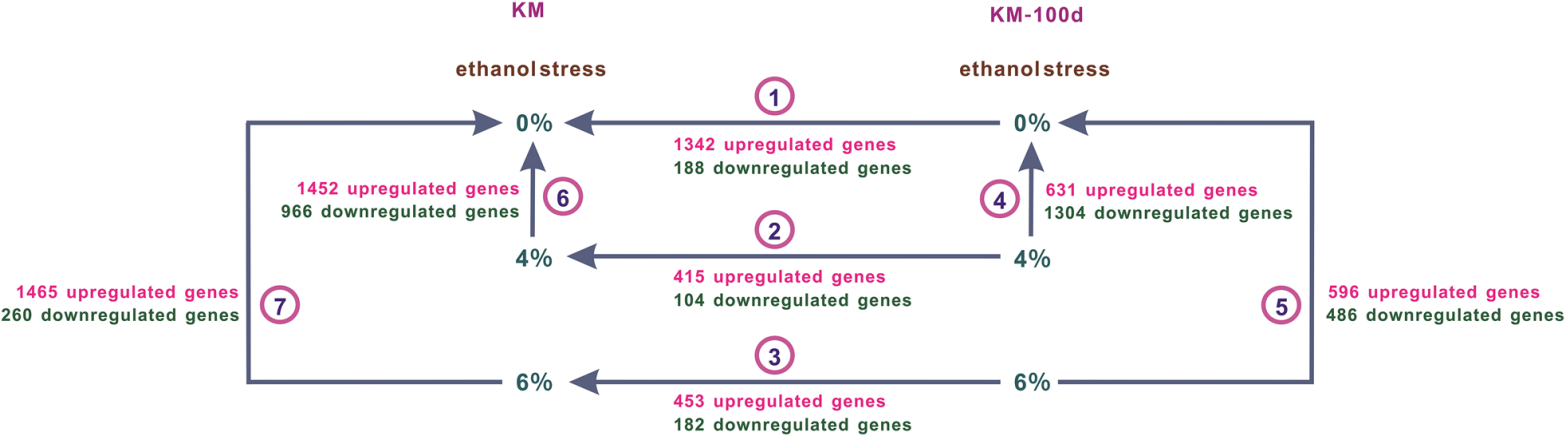
Global analysis of RNA-seq data in KM and KM-100d under ethanol stress. In this figure, we compartmentalized RNA-seq data for gene differential expression analysis. KM and KM-100d were presented in the left and right parts, respectively, and ethanol concentrations 0%, 4%, and 6% (v/v) were located in rows. RNA-seq data were divided into seven groups, denoted as ①, ②, ③, ④, ⑤, ⑥, and ⑦. In each group, an arrow points to the control for differential expression identification, and the red digits denote the up-regulated gene number, while the green ones represent the down-regulated number

To thoroughly understand the ethanol tolerance, we performed an RNA-seq analysis to compare gene expressions of the KM and KM-100d yeasts that grew in media with 4% and 6% (v/v) ethanol. Based on the growth profiles (Fig. 1c), it was observed that growth ability between KM-100d and KM had the maximum difference at 48 h; hence, samples at 48 h were collected for RNA-seq analysis. Setting |log_2_ratio| ≥ 1 and *p* value < 0.05 as the criterion for defining significant Differentially Expressed (DE) genes, we performed DE gene identification in seven groups (Fig. 2, denoted as 1, 2, 3, 4, 5, 6, and 7, respectively). In each group, DE analysis was carried out according to the arrow pointing to the control. Additional file 3 provides expression values and differential expression statistics of all genes. There is a global expression difference between KM and KM-100d when both of them grew in an ethanol-free medium (Fig. 2 Group ①). KM-100d yeasts have 1342 genes with higher expression than that of the KM yeast, while only 188 genes have lower expression. In media with 4% and 6% (v/v) ethanol, the numbers of up-regulated genes in KM-100d are greatly reduced to 415 (Group ②) and 453 (Group ③), respectively. However, the up-regulated gene numbers are still much more than those with lower expression (104 and 182 genes, respectively). Those results suggest that KM-100d yeasts are transcriptionally rewired by activating a great number of genes. The suggestion was further supported by the ethanol-induced expression changes within the KM and KM-100d yeasts. Under induction of 4% and 6% (v/v) ethanol, the KM yeasts have 1452 and 1465 up-regulated genes (Groups ⑥ and ⑦) while the KM-100d yeasts have only 631 and 596 up-regulated genes (Groups ④ and ⑤), respectively. In addition, we applied heatmap.2 software in R package to cluster genes and groups based on log_2_ratio values (Additional file 1: Figure S3) and found the gene differential expression profile in Group ① is close to that of Group ⑦. Taken together, the KM-100d yeasts maintained many ethanol-induced expression features even if they grew in an ethanol-free medium. The features make the evolved cell being more adaptive to up-coming ethanol stimulation, i.e. KM-100d yeast needn’t activate as many genes as KM does.

Subsequently, in each group, we performed gene ontology (GO) enrichment analysis for DE genes (Additional file 1: Figure S4), and found that the enriched GO terms covered a wide range of cellular basic physiological processes, including ribosome biogenesis, amino acid biosynthesis, DNA repair, RNA processing, etc., but not directly relevant to ethanol resistance. Therefore, in the following, we particularly focused on pathways strongly associated with ethanol metabolism and tolerance, by analyzing the associated DE genes (Additional file 4).

### KM-100d enhanced ethanol usage

**Fig. 3:**
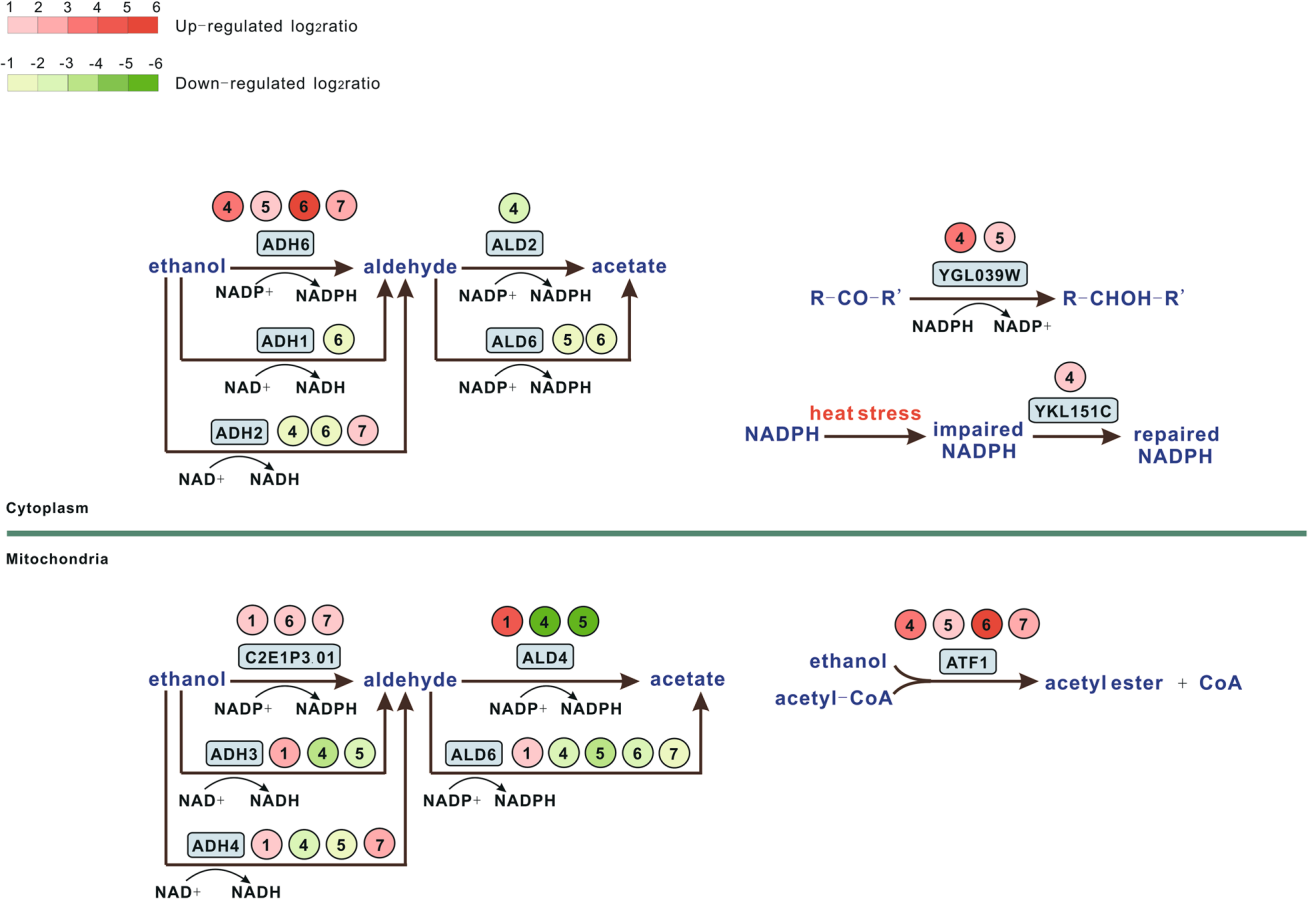
Possible ethanol consumption routes in *K. marxianus*. In this figure, ethanol is consumed both in the cytoplasm (the upper part) and mitochondria (the lower part). Blue–gray rectangle denotes the involved gene, and Groups ①, ②, ③, ④, ⑤, ⑥, and ⑦ are in line with those definitions in Fig. 2. Red and green in group number denote gene’s up- and down-regulation, respectively. Color’s intensity represents the degree of gene expression change, the correspondence of color and log_2_ratio value is quantified by the color bar in figure’s top left corner

To facilitate graph illustration, the log_2_ratio values of involved DE genes (Additional file 4) were subdivided into five intervals: 1 ~ 2, 2 ~ 3, 3 ~ 4, 4 ~ 5, 5 ~ 6 (and above), as shown in Fig. 3.

The difference between KM and KM-100d in ethanol consumption was investigated. As illustrated in Fig. 3, two routes may exist for directly consuming ethanol, and were both up-regulated in KM and KM-100d when faced ethanol (Groups ④, ⑤, ⑥, and ⑦). One way is via cytoplasmic ADH6, which catalyzes ethanol to acetaldehyde, facilitated by NADP+. The other is by mitochondrial ATF1, which promotes ethanol esterification with the aid of acetyl-CoA. Especially, in KM-100d under ethanol stress (Groups ④ and ⑤), YGL039W was up-regulated to generate more NADP+, and thus may supply more coenzymes for converting ethanol into acetaldehyde to reduce ethanol toxicity. In mitochondria, for KM exposed in ethanol stress (Groups ⑥ and ⑦), only C2E1P301 and ADH4 were up-regulated. While in KM-100d, during the process from ethanol to aldehyde to acetate, C2E1P301, ALD4, ADH3, ADH4, and ALD6 were all up-regulated (Group ①). The aforementioned changes indicate that KM-100d might acquire novel capability to alleviate ethanol toxicity by increasing ethanol consuming. The theory explains that KM-100d performed over KM in medium with ethanol.

### KM-100d enhanced membrane lipid biosynthesis, anti-osmotic pressure, anti-oxidative stress, and protein folding to resist ethanol stress

**Fig. 4:**
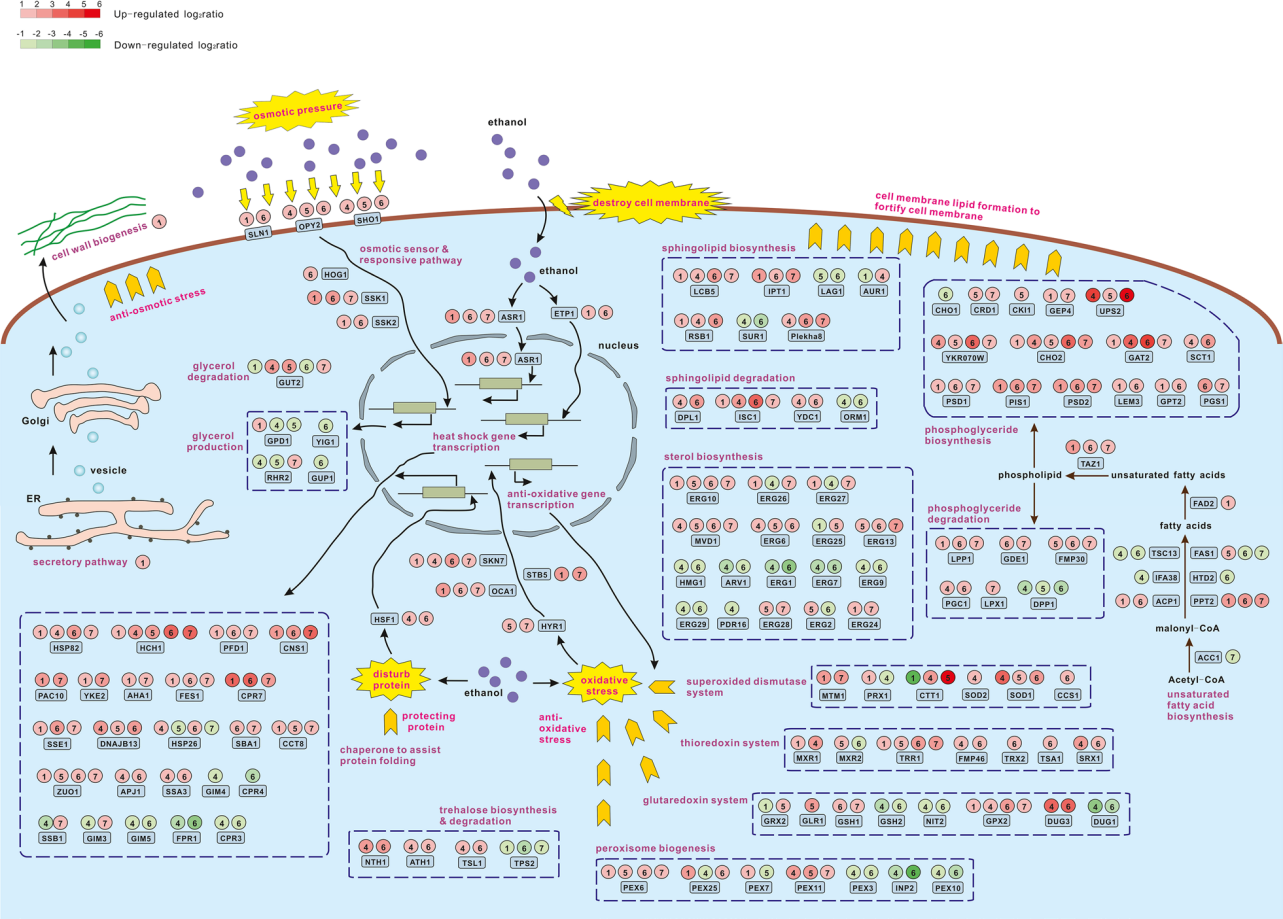
A schematic diagram for anti-ethanol caused damage in *K. marxianus*. In the left part of this figure, accumulated ethanol in the medium imposes an osmotic pressure to the yeast cell and subsequently activates the osmotic responsive pathway. In the middle part, environmental ethanol permeates into the cell and disrupts cell membrane. In the right part, cell membrane lipids are synthesized to fortify and repair the damaged cell membrane. In the lower part, ethanol disturbs protein conformation and causes oxidative stress, thus the related sensors and response pathways are activated. The group numbers and colors for the gene’s differential expression are in line with those in Fig. 3

Besides being consumed, the accumulated ethanol directly influences cell membrane integrity, alters inner- and outer-osmotic pressure [3], disturbs protein conformation [3, 32], and induces reactive oxygen species (ROS) generation [33], thereby causing serious damage to yeast cell [34]. In the following, we analyzed DE genes in these pathways for anti-ethanol-caused damages in *K. marxianus* (Fig. 4).

After ethanol-driven evolution, the alcohol stress response pathway in KM-100d was activated. ASR1, which translocates from the cytoplasm to the nucleus upon exposure to ethanol [35], and ETP1, which acts as a cytoplasmic retention protein with a role in ethanol-dependent transcriptional activation of heat shock protein genes [36], were both up-regulated in KM-100d (Group ①), and partly up-regulated in KM faced ethanol (Groups ⑥ and ⑦), suggesting even in an ethanol-free medium, KM-100d may prepare responsive pathways for ethanol challenge, just like KM’s response to ethanol.

The formation of membrane lipid plays an important role in keeping cell membrane integrity under ethanol stress [18]. Unsaturated fatty acids, the key components in cell membrane structure, are closely related to ethanol tolerance [37]. Illustrated in Fig. 4, from acetyl-CoA to unsaturated fatty acid biosynthesis to incorporation into phospholipid, the involved genes PPT2, FAD2, and TAZ1 were all up-regulated in KM-100d (Group ①), suggesting that after evolution, KM-100d may already enhance unsaturated fatty acid biosynthesis to supply more raw materials for cell membrane biogenesis. And the down-regulation of genes ACC1, TSC13, FAS1 in KM exposed in ethanol (Groups ⑥ and ⑦), is accordant with the previous report [28], which may account for *K. marxianus*’ weak ethanol tolerance before adaptive evolution.

A large number of genes associated with cell membrane lipid biogenesis, including phosphoglyceride, sphingolipid, and sterol, were differentially expressed under ethanol stress (Fig. 4). Especially, genes involved in phosphoglyceride biosynthesis were generally up-regulated in KM-100d (Group ①) and in KM-faced ethanol (Groups ⑥ and ⑦), indicating that phosphoglyceride may be the major component in cell membrane for ethanol resistance. For sterol biosynthesis, many genes (e.g., ERG9 and ERG7) were down-regulated in Groups ④ and ⑥, and several genes were up-regulated in Group ⑤(e.g., ERG25) and Group ⑦ (e.g., ERG26), suggesting that sterol biogenesis may be important for standing up to high ethanol, but not for low ethanol. In addition, genes CKI1, ERG2, and ERG25, involved in phosphoglyceride and sterol biosynthesis, were only up-regulated in Group ⑤; this may be one of the clues for KM-100d’s better performance than KM in high ethanol.

High ethanol concentration in a medium imposes osmotic stress on yeast cells [2, 3]. As shown in Fig. 4, the plasma membrane osmotic sensors (SLN1, OPY2, and SHO1), and genes (HOG1, SSK1, and SSK2) involved in the downstream responsive pathway were all up-regulated in KM exposed in low ethanol (Group ⑥), and individually up-regulated in KM-100d (Group ①), in KM-100d faced ethanol (Groups ④ and ⑤), and in KM confronted high ethanol (Group ⑦). Glycerol production is a major way for *S. cerevisiae* resisting osmotic pressure [38]. When KM and KM-100d faced ethanol, most genes related to glycerol biogenesis were down-regulated. The above findings suggest that before and after evolution, *K. marxianus* always enhance pathways for sensing and transducing osmotic stress signals; however, its strategy for resisting osmotic stress may not rely on the glycerol production route, there must exist some other ways.

Cell wall provides sufficient mechanical strength for the cell to withstand osmotic pressure [39], and the secretory pathway not only transports cell wall proteins outwards but also transfers lipids onto plasma membrane to fortify membrane structure; therefore, these two processes may be responsible for anti-osmotic stress. We analyzed DE genes in the secretory pathway and cell wall biogenesis (Additional file 1: Figure S5). Genes involved in the secretory pathway and cell wall biogenesis were generally up-regulated in KM-100d (Group ①), and some of them were also up-regulated in KM exposed in ethanol (Groups ⑥ and ⑦). It implies that KM-100d not only maintained the up-regulation in secretory pathway for KM resistant to ethanol stress but also widely enlarged the activation scope of secretory pathway to prepare for ethanol attack; hence, enhancement of secretory pathway and cell wall formation may be the new strategy KM-100d developed to endure ethanol-caused osmotic stress. On the other hand, for KM and KM-100d exposed in low ethanol (Groups ④ and ⑥), many genes in the secretory pathway were both down-regulated, suggesting that secretory pathway activation may not be the necessary way for *K. marxianus* responding to low ethanol.

For against the oxidative stress triggered by ethanol (Fig. 4), HYR1, which functions as a sensor and transducer of hydroperoxide stress [40], was up-regulated in KM and KM-100d both exposed in high ethanol (Groups ⑤and ⑦). Oxidative stress-responsive transcription factors SKN7 and STB5 were up-regulated in KM-100d (Group ①) and in KM faced high ethanol (Group ⑦). For anti-oxidative stress, genes involved in the superoxide dismutase system (e.g., MTM1 and PRX1) were generally up-regulated in KM-100d either exposed in ethanol or not (Groups ①, ④ and ⑤). For the thioredoxin system, genes (e.g., MXR1 and TRR1) were up-regulated in KM and KM-100d both faced ethanol. Thioredoxin and its reductase were also reported to enhance *K. marxianus* tolerance to multiple lignocelluloses-derived inhibitors [41]. For the glutaredoxin system, genes GRX2 and GLR1 were only up-regulated in KM-100d exposed in high ethanol (Group ⑤). For peroxisome biogenesis, genes such as PEX6 and PEX7 were up-regulated in KM-100d (Group ①), and some other genes (e.g., PEX3 and INP2) were down-regulated in KM and KM-100d when faced low ethanol (Groups ④ and ⑥). The above implies that KM-100d may globally strengthen anti-oxidation ability.

To ensure proper protein folding under ethanol stress (Fig. 4), the heat shock transcription factor HSF1 was up-regulated in KM and KM-100d faced low ethanol (Groups ④ and ⑥), a number of chaperone-related genes (e.g. PFD1 and CPR7) were up-regulated in KM faced ethanol (Groups ⑥ and ⑦), also kept up-regulation in KM-100d (Group ①). Some genes (e.g. CPR4) down-regulated in KM faced ethanol were not down-regulated in KM-100d. Therefore, KM-100d may generally enhance protein folding to provide a more stable cellular environment.

It was reported that in *S. cerevisiae*, trehalose accumulation was important for ethanol tolerance, due to trehalose working as compatible solute to prevent influx of excess salts into yeast cells [3]; meanwhile, genes involved in trehalose degradation were also induced by ethanol, to adjust it at the optimal concentration [3]. For *K. marxianus* (Fig. 4), we found that genes participating in trehalose biosynthesis and degradation (e.g., NTH1 and TSL1) were commonly up-regulated when treated with low ethanol (Groups ④ and ⑥), but not gene up-regulated in trehalose metabolism when exposed in high ethanol (Groups ⑤ and ⑦). This suggests that in *K. marxianus*, trehalose accumulation may be a special strategy for coping with low ethanol, but not for high ethanol.

**Fig. 5:**
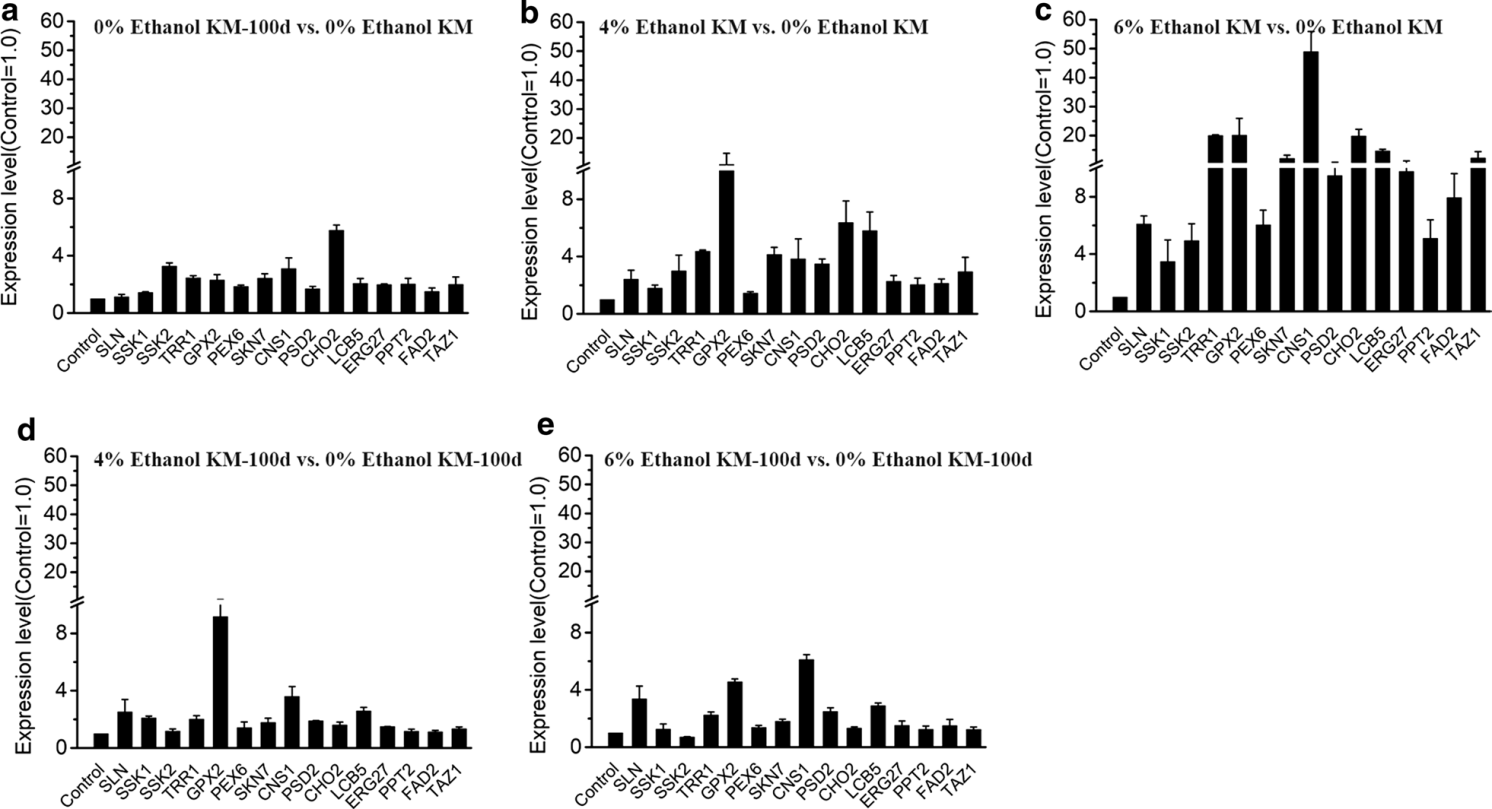
RT-qPCR analysis for gene differential expression of KM and KM-100d in different groups. **a** KM-100d vs. KM both in the ethanol-free medium. This is for DE gene analysis in Group ①. **b** KM exposed in 4% (v/v) ethanol vs. KM in an ethanol-free medium. This is for Group ⑥. **c** KM exposed in 6% (v/v) ethanol vs. KM in an ethanol-free medium. This is for Group ⑦. **d** KM-100d exposed in 4% (v/v) ethanol vs. KM-100d in an ethanol-free medium. This is for Group ④. **e** KM-100d exposed in 6% (v/v) ethanol vs. KM-100d in an ethanol-free medium. This is for Group ⑤. In each sample, 18S was used as an internal control. Total RNA was isolated from cells cultured at 48 h in biological triplicate

The differential expressions of 15 genes involved in the above ethanol-tolerant pathways were validated with RT-qPCR analysis (Fig. 5). Genes’ expressions in Group ① (Fig. 5a) were generally up-regulated. On the other hand, the up-regulation of genes in Group ④ (Fig. 5d) and Group ⑤ (Fig. 5e) was not as high as in Group ⑥ (Fig. 5b) and Group ⑦ (Fig. 5c). Our analysis confirmed that genes contributing to ethanol tolerance are continuously activated in KM-100d even after the withdrawn of ethanol stress. The continuous gene expression might be helpful to stable the intracellular environment and benefit to growth of cells in upcoming stress.

### Validation of enhanced ethanol consumption and multiple-stress resistance in KM-100d

**Fig. 6:**
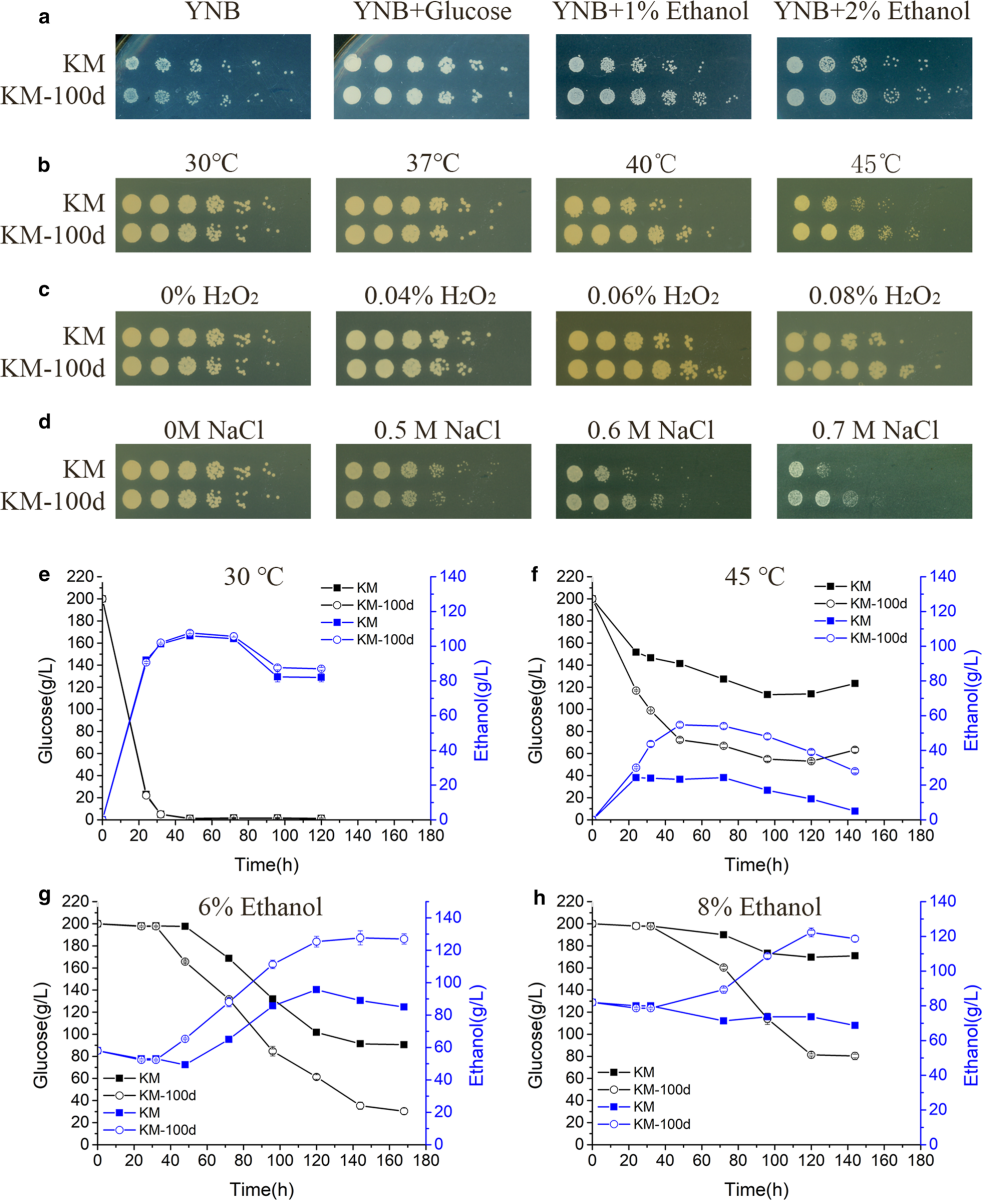
Cell growth assay on ethanol and cell viability and fermentation under multiple stresses. **a** Cell growth assay of KM and KM-100d based on glucose or ethanol as a carbon source. A cell suspension of 10 OD_600_ was fivefold serially diluted and spotted onto a YNB plate with glucose or ethanol as the only carbon source and cultured for 2 days, as illustrated along columns. **b** Cell viability assay of KM and KM-100d under thermic stress. The temperatures are 30 °C, 37 °C, 40 °C, and 45 °C. **c** Cell viability assay under oxidative stress. H_2_O_2_ was used as oxidation stimulus, and its concentrations are 0%, 0.04%, 0.06%, and 0.08%. **d** Cell viability under osmotic pressure. High salt (NaCl) was used to cause osmotic pressure, with concentrations of 0 M, 0.5 M, 0.6 M, and 0.7 M. **e** Ethanol yield and glucose utilization in KM and KM-100d in the basic circumstance. **f** Ethanol yield and glucose utilization under 45 °C. **g** Ethanol yield and glucose utilization under 6% (v/v) ethanol stress. **h** Ethanol yield and glucose utilization under 8% (v/v) ethanol stress. In subfigure **b**–**d**, KM and KM-100d are in the first and second rows, respectively. A cell suspension of 10 OD_600_ was 5-fold serially diluted and spotted onto a YPD plate and cultured for 2 days. Except for the thermic test with specified temperatures, other tests were all carried out at 30 °C. In subfigure **e**–**h**, the left *y*-axis and right *y*-axis represent glucose residues in the medium (denoted in black) and ethanol production (denoted in blue), respectively

We checked the growth of KM and KM-100d yeasts in YNB medium with different carbon sources (Fig. 6a). Both the KM and KM-100d yeasts have the same capability to utilize glucose as the only carbon source. However, in the presence of 1% and 2% (v/v) ethanol as the sole carbon source, KM-100d yeasts grew better than KM yeasts. The result supports our aforementioned hypothesis that the KM-100d yeasts utilized ethanol more efficiently than the KM yeasts did.

On the other hand, based on Fig. 4, KM-100d may develop resistance to ethanol-caused multiple stresses simultaneously, including osmotic stress, oxidative stress, and thermic stress (for heat shock proteins taken as chaperones for protein folding). To evaluate the yeasts’ tolerances to multiple stresses, we conducted the cell viability assay for various temperature, oxidation, and osmotic pressures, respectively (Fig. 6b–d). In the basic circumstance (i.e., 30 °C, 0% H_2_O_2_, and 0 M NaCl), a difference of viability is lacking between the KM and KM-100d yeasts. However, in the presence of the different stresses, KM-100d yeasts exhibit significantly better viability than that of the KM yeasts. Further, the advantages are more significant while the stresses became more serious (Fig. 6b–d). We expected that the tolerances to multiple stresses might introduce new features to the yeasts in ethanol production.

**Fig. 7:**
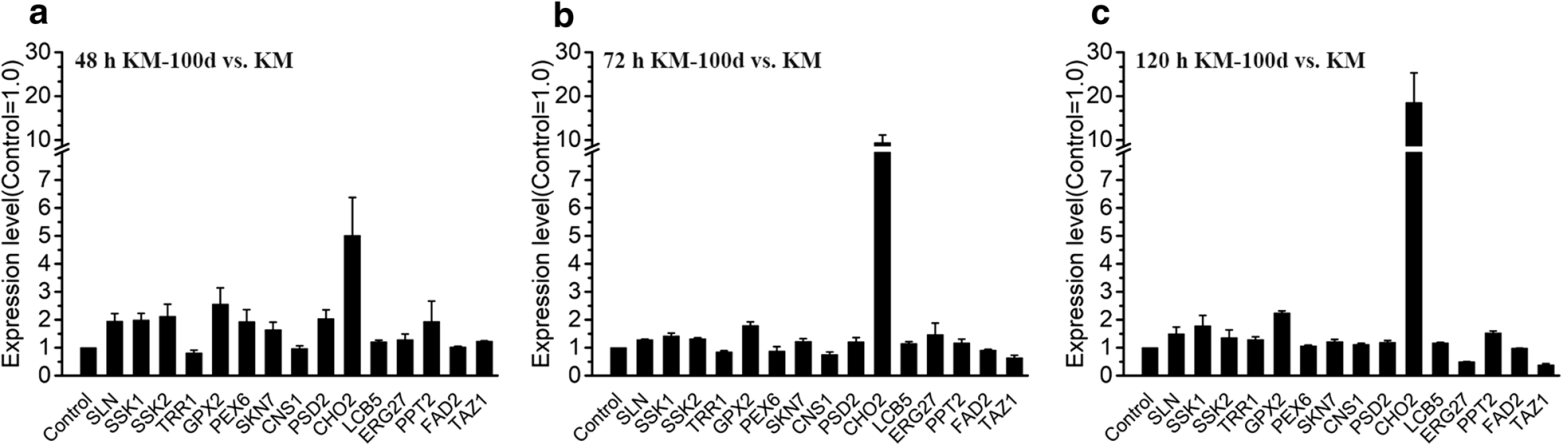
RT-qPCR analysis for gene expressions in KM and KM-100d during fermentation. **a** KM-100d vs. KM both at 48 h in fermentation. **b** KM-100d vs. KM both at 72 h in fermentation. **c** KM-100d vs. KM both at 120 h in fermentation. In each sample, 18S was used as an internal control. Both KM and KM-100d were cultured in media with 6% ethanol. Total RNA was isolated from cells cultured in biological triplicate

We further investigated ethanol productivity between the KM and KM-100d yeasts under multiple stresses. In basic circumstance (30 °C and 0% ethanol, Fig. 6e), the KM and KM-100d yeasts are nearly the same in both glucose consumption and ethanol production. However, the KM-100d yeasts showed significant advantages in the presence of high temperature (45 °C, Fig. 6f) or more ethanol (6% or 8%, Fig. 6g, h); the common environment usually happened at the late stage of fermentation. We conducted an RT-qPCR analysis for both KM and KM-100d yeasts fermented in media with 6% ethanol at 48 h, 72 h, and 120 h (Fig. 7). Most of these ethanol-tolerant genes were up-regulated in KM-100d compared to in KM, especially at the earlier stage (48 h) for initial adjustment to ethanol (Fig. 7). To sum up, by up-regulating expressions of ethanol-tolerant genes, the KM-100d yeasts outperformed the KM yeasts under stressful environments with increased ethanol yields.

## Discussion

After adaptive experimental evolution in 6% (v/v) ethanol for 100 days, the maximum tolerable ethanol concentration for *K. marxianus* was elevated up to 10% (v/v). By RNA-seq analysis, we found that KM-100d’s regulatory network had been genome-wide rewired. Compared to KM, even cultured in an ethanol-free medium, KM-100d improved key pathways closely related to ethanol resistance, covering ethanol usage, ethanol sensing, membrane lipid formation, anti-osmotic pressure, anti-oxidative stress, and protein folding, as prepared to alleviate the possible intracellular damage caused by upcoming ethanol attack.

In the studies of *S. cerevisiae* adaptive laboratory evolution under ethanol stress, not only mutations occurred in the genome [25, 26], but also the genome ploidy changed; diploid was the stable ploidy for *S. cerevisiae* resistant to ethanol [25]. Further for *S. cerevisiae* strains with different genome ploidy propagating in 2 different evolutionary environments, an unstressful environment and a salt-stressed environment, they both converged toward diploidy [42]. In contrast, we found that after the haploid *K. marxianus* evolution in ethanol for 100 days, the obtained KM-100d population had no ploidy change (Additional file 1: Figure S2), suggesting that haploid may be the stable ploidy for *K. marxianus* tolerant to ethanol, and this is in line with its common natural occurrence as haploid isolated from non-dairy environments [43, 44]. By DNA-seq analysis of KM and KM-100d, only 4 SNP mutations took preponderance in KM-100d population (Additional file 2); 3 of them were in genes but lacked meaningful alteration. These suggest that in *K. marxianus*, ethanol tolerance improvement may be majorly based on transcriptional reprogramming rather than DNA context alterations. Further RNA-Seq analysis revealed that many genes involved in epigenetics, including histone acetylases, were up-regulated in KM-100d (Group ①), which may result in the global activation of gene expressions and phenotypic change in KM-100d. Likewise, in the researches of antibiotic tolerance of bacteria[45], cells can develop drug tolerance based on a change in bacterial physiology, instead of genetic change. Therefore, tolerant state arising from transcriptional rewiring rather than genetic variation may be universal in nature.

By RNA-seq analysis, the pathways of cytoplasmic protein folding, anti-osmotic pressure, and anti-oxidative stress were all enhanced in KM-100d (Fig. 4), which are similar to *S. cerevisiae* resistant to ethanol [3, 16, 46, 47]. Interesting, we further confirmed that KM-100d developed a novel multi-stress-resistant capability, i.e., resilient to ethanol, high temperature, oxidative stress and osmotic stress, which may be due to the multi-aspect damage caused by ethanol. Similarly, a recent study found that after *S. cerevisiae* adaptive evolution in acetic acid, improved tolerance to multiple stresses was gained, and the stress cross-tolerance could be explained by its enzymatic antioxidative capacity [27]. The above findings suggest that yeast can simultaneously develop resistances to multiple stresses with only one selective pressure because of shared pathways in the multiple resistances.

There are still some differences between the ethanol tolerance of *K. marxianus* and *S. cerevisiae*. (1) Consuming the ethanol diffused from the medium should be one of the first responses for ethanol attack. However, little report has referred this. In this study, by RNA-seq analysis (Fig. 3), we found KM-100d may improve consumption on ethanol, and this prediction was further validated by cell growth assay fed on ethanol (Fig. 6a). (2) For cell membrane lipid biogenesis, genes involved in sterol biosynthesis were down-regulated under ethanol stress in *S. cerevisiae* [47, 48], but in KM-100d, the biosynthesis of phosphoglyceride, sphingolipid, and sterol were all strengthened (Fig. 4). (3) For resisting the osmotic stress caused by ethanol, glycerol production was the major way in *S. cerevisiae* [38], while in KM-100d, it may be mainly via activating secretory pathway and cell wall formation to stand up to osmotic stress (Fig. 4, Additional file 1: Figure S5). Notably, secretory pathway transports cell wall proteins onto plasma membrane, as well as transfers lipids via vesicles to repair cell membrane damaged by ethanol, and thus may be essentially responsible for ethanol tolerance, but has not been mentioned in *S. cerevisiae*.

It should be noted that, for KM and KM-100d responding to low ethanol (Groups ④ and ⑥), many genes have common expression changes, i.e., both were up-regulated or down-regulated. For instance, genes APJ1 and SSA3 involved in protein-folding (Fig. 4) were both up-regulated in Groups ④ and ⑥, while genes related to sterol biosynthesis (e.g. ERG9 and HMG1 in Fig. 4) and genes participating in ER to Golgi vesicle transport (e.g. SEC13 and SEC16 in Additional file 1: Fig. S3), were both down-regulated in Groups ④ and ⑥. In contrast, for KM and KM-100d responding to high ethanol (Groups ⑤ and ⑦), little commonality for gene expression change is shown. The strong commonality between Groups ④ and ⑥, and weak commonality between Groups ⑤ and ⑦, suggest that KM-100d is evidently different from KM especially at high ethanol; thus, a memory for the adaptive evolution under 6% (v/v) ethanol may exist.

Interestingly, *K. marxianus* seemed to start up distinct routes for resisting to different ethanol concentrations. For facing low ethanol (Groups ④ and ⑥), KM and KM-100d particularly set up trehalose metabolism to protect cells (Fig. 4); and for bearing high ethanol (Groups ⑤ and ⑦), sterol biosynthesis (Fig. 4) and the whole secretory pathway (Additional file 1: Fig. S3) were activated. Based on the above, we conjecture that when ethanol concentration is low, cell membrane may be only mildly impaired, and cytoplasmic trehalose accumulation might be sufficient for this case; while when environmental ethanol is as high as dangerous to cell survival, plasma membrane may be severely damaged; thus, sterol biosynthesis and secretory pathway have to be activated to assist membrane repair, even though these processes demand much reducing power for lipid biosynthesis and a lot of energies for vesicle transport.

In addition, KM-100d has the same ethanol production as KM in a basic circumstance (Fig. 6e), but in the existence of ethanol or thermic stress, KM-100d provides much better ethanol production than KM does (Fig. 6f–h). The better production may be due to the improved cell survival in the stressful circumstances. This finding could remedy the previously reported disappointing data about *S. cerevisiae* ethanol production through adaptive laboratory evolution under ethanol stress [25, 38, 49]. In these studies, ethanol yields were either not reported [25], or not elevated [38], or even decreased [49]. These data are suggested to be re-measured under ethanol stress, which is more approximate to the realistic industrial environment, and some exciting data may occur.

## Conclusions

The wild-type *K. marxianus* was conducted in an adaptive evolution driven by 6% (v/v) ethanol for 100 days, resulting in ethanol tolerance increased up to 10% (v/v). RNA-seq analysis of the pre- and post-evolution *K. marxianus* found that even in an ethanol-free medium, KM-100d generally maintained up-regulation of many pathways closely related to ethanol resistance, as if always prepared for the upcoming ethanol attack. In addition to ethanol tolerance, KM-100d may also develop resistance to other stresses, including thermic, osmotic, and oxidative stresses, and was confirmed by cell viability assay. And the improved tolerance led to increased ethanol production in stressful circumstance. Our study may give rise to routes for rational improvement of *K. marxianus*’ ethanol tolerance, and may also provide desirable candidate strains for industrial bioethanol production.

## Methods

### Yeast strains

The *K. marxianus* strain used for experimental adaptive evolution was derived from the wild-type haploid *K. marxianus* strain FIM1 (*CGMCC No. 10621*), which was deposited at China General Microbiological Culture Collection Center (CGMCC) with a reference number of 10621.

### Experimental evolution

*Kluyveromyces marxianus* cells from a single colony were inoculated in 15-mL YPX medium (1% yeast extract, 2% peptone, and 2% xylose) without any antibiotic supplement and incubated at 30 °C in an orbital shaker under 220 rpm for 24 h. Then, the cells were subcultured into a new flask containing 15-mL fresh medium (1% yeast extract, 2% peptone, 2% xylose, and 6% v/v ethanol) to start a transferring cycle at initial OD_600_ of 0.6. After 100 days of the transfer, cells were stored in 20% glycerol at − 80 °C for subsequent experiments. During the whole course of adaptive evolution, OD_600_ was measured daily to record cell growth status. The original *K. marxianus* yeast before evolution is termed as KM, and the 100-day evolved *K. marxianus* population is termed as KM-100d in this study.

### Cell growth profiling

For comparing cell growth of KM and KM-100d under different ethanol concentrations, strains were previously inoculated in 50-mL YPX medium overnight under agitation at 220 rpm at 30 °C, and then grown into a new flask containing 50-mL fresh medium (1% yeast extract, 2% peptone, and 2% xylose) with different ethanol concentrations at 0, 4, 5, 6, 7, 8% (v/v), at initial OD_600_ of 0.6. After that, 150-μL samples were collected into sterile 96-well plates every 2 h. Before each measurement, cell cultures were automatically shaken for 180 s to homogenize the samples, and values were measured at the length of 600 nm. Each test was performed in biological triplicate. Then, the OD_600_ average of triplicate was calculated and cell growth profile was plotted accordingly.

### Estimation of tolerance to multiple stresses

We estimated the ethanol tolerance of KM and KM-100d with the modified method proposed by Ogawa et al. [50]. Cells from KM-100d and KM were first inoculated on a YPD plate and cultured at 30 °C overnight. Then, cells on the plate were moved to 20-mL YPD liquid medium and cultured at 30 °C under 250 rpm for 24 h to ensure cells reaching the stationary phase. Afterwards, cells were centrifuged at 8000 rpm for 30 s and supernatant was removed. Cells suspended in 1-mL sterilized water were centrifuged and harvested. The cells harvested were subsequently resuspended in sterilized water to 10^5^–10^6^ cell/μL. The cell suspensions of 10 μL was added into 5 mL medium with 0.1 M acetate buffer (pH 5.5), 1% glucose, and different ethanol (0, 1, 2,…, 11%, v/v). The cultures were incubated at 30 °C under 250-rpm shaking for 3 days. A cell suspension of 5 μL from the liquid medium was serially diluted by fivefold and spotted onto a YPD plate and cultured at 30 °C for 48 h.

To estimate cell viability under different concentrations of H_2_O_2_, overnight-cultivated cells were collected and adjusted to an OD_600_ of 0.5. Samples were diluted by fivefold for five times. Dilutions were spotted onto YPD medium containing different concentrations of H_2_O_2_ (0%, 0.04%, 0.06%, and 0.08%). To estimate cell viability under different osmotic pressure, collected cells were spotted onto YPD medium containing different concentrations of NaCl (0 M, 0.5 M, 0.6 M, and 0.7 M). For estimation of temperature resistance of KM and KM-100d, collected cells were spotted onto YPD medium and then cultured at different temperatures (30 °C, 37 °C, 40 °C, and 45 °C). All the above plates were incubated for 2 days before imaging.

### Ethanol utilization tests

Ethanol utilization tests were carried out in YNB plates containing 6.7 g/L yeast nitrogen base (YNB) and 2 g/L glucose or ethanol individually. Overnight-cultivated cells were collected and adjusted to an OD_600_ of 0.1. Samples were diluted by foutfold for five times. Dilutions were spotted onto YNB medium containing different carbon sources. Plates were incubated for 2 days before imaging.

### Shaking flask fermentation

KM and KM-100d were transferred from YPD plates to 150-mL Erlenmeyer flask containing 50 mL of YPD broth. Yeasts were inoculated for overnight at 220 rpm on a rotary shaker at 30 °C. Yeasts were transferred to each 150-mL Erlenmeyer flask containing 50 mL of the fermentation medium containing 200 g/L glucose to make an initial concentration of 0.6 OD_600_. We also added 6% or 8% ethanol to test the yeast fermentation ability under ethanol pressure. The Erlenmeyer were shaken at 200 rpm and 30 °C or 45 °C. Samples were taken at an interval of 12 h for SBA-40D biosensor analyzer (Baisheng, Jinan, China). Fermentation experiments were conducted in biological triplicate.

### RT-qPCR analysis

The expression levels of ethanol tolerance related genes were determined by real-time reverse transcription PCR (RT-qPCR). To observe these gene expressions in Groups ①, ②, ③, ④, ⑤, ⑥, and ⑦, KM and KM-100d were inoculated at 30 °C in 50-mL YPD media containing 0%, 4%, 6% ethanol at 220 rpm, respectively, and total RNA from cells cultured at 48 h in biological triplicate was isolated with quick-RNA fungal/bacterial miniprep kit (ZYMO RESEARCH. Beijing, China). And to evaluate gene transcription changes during the fermentation in the ethanol-tolerance-increased case, KM and KM-100d were transferred from YPD plates to 50 mL of YPD medium. Yeasts were inoculated for overnight at 220 rpm at 30 °C. Then, yeasts were transferred to 50 mL of the fermentation medium containing 200 g/L glucose, 20 g/L peptone, 10 g/L yeast extract and 6% ethanol at an initial concentration of 0.6 OD_600_. Total RNA from cells cultured at 48 h, 72 h and 120 h in biological triplicate were used to do RT-qPCR.

The total RNA was quantitatively determined by Nanodrop 2000 (Thermo Fisher, Massachusetts, USA). cDNA was synthesized by PrimeScript RT reagent kit (Takara. Beijing, China). Reverse transcription reaction was performed on a thermal cycler (Applied Biosystems, USA) at 37 °C for 15 min, 85 °C for 5 s. Real-time PCR was conducted on an Applied Biosystems 7900/HT (Applied Biosystems, USA) with TB green premix EX TaqII (Takara. Beijing, China). 18S was used as an internal control. The primers for RT-qPCR were listed in Additional file 1: Table S1.

### Sample preparation for DNA-seq and RNA-seq

Cells taken from each replicate incubation at 48 h were collected in pre-chilled Corning tubes and were centrifuged at 4 °C for 2 min; then, the cell pellets were stored at − 80 °C before analysis. Total DNA was extracted using the Yeast Genomic DNA Extraction kit (Solarbio, China) and total RNA was extracted using the ZR Fungal/Bacterial RNA MiniPrep™ (Zymo Research, CA). The samples were then sent to the Institute of Biochemistry and Cell Biology (Shanghai, China) for quality and quantity evaluation and sequencing. Samples for RNA-seq and DNA-seq investigation were both in biological triplicate.

### DNA-seq analysis

After initial QC, short 150 bp reads for genomic DNA were mapped to the reference genome of *K. marxianus* DMKU 3-1042 [30] using HISAT2.1 [51]. The average coverage is 695 X and 654 X for the KM-100d and KM yeasts, respectively. The SAM files were transformed into BAM format in samtools 1.7 [52]. The sequencing data of KM-100d and KM yeasts were analyzed in SomaticSniper (v1.0.5.0) [53] to find out possible genetic alternatives. The fastq DNA-seq data have been deposited in the Genome Sequence Archive (GSA) server at the BIG Data Center in Beijing Institute of Genomics (http://bigd.big.ac.cn, Bioproject accession No. PRJCA001291 and GSA accession No. CRA001456).

### RNA-seq analysis

We obtained 15.1 million pair-end reads on average for each of the RNA samples. After initial QC, short 150 bp reads were mapped to the reference genome of *K. marxianus* DMKU 3-1042 [30] using HISAT2.1 [51]. The alignment rates are in a range from 86 to 94% for different RNA samples. The differential expression analysis for the RNA-seq data was first conducted in edgeR (v3.8). The initial results showed a global expression difference that might impact the normalization method (Trimmed Mean of M-values) of the edgeR package, which is based on the hypothesis that most genes are not DE. In a further comparison of gene expression, we conducted a normalization using the total count of reads as a straightforward approach. In this study, for gene expression difference analysis, we presented *p* values from edgeR but fold changes from the straightforward normalization.

## Additional files

**Additional file 1: Fig. S1.** The growth rate of KM and KM-100d at different ethanol concentrations. **Fig. S2.** Flow cytometer analysis for DNA content variation during *K. marxianus* evolution. **Fig. S3.** Heat map for log_2_ratio values with clustered genes and groups. **Fig. S4.** GO enrichment analysis for KM and KM-100d exposed in low and high ethanol. **Fig. S5.** Secretory pathway and cell wall biogenesis in *K. marxianus* pre- and post-evolution. **Table S1.** Primers for RT-qPCR analysis.

**Additional file 2.** DNA mutations in KM-100d compared to KM. A file containing SNP mutation sites and frequency in KM-100d population.

**Additional file 3.** Transcriptome data and differential expression analysis. A file containing the expression data of all genes in *K. marxianus* based on RNA-seq analysis, as well as their differential expression analysis results, according to the groups defined in Fig. 2.

**Additional file 4.** Genes involved in pathways closely related to ethanol tolerance. A file containing the referred pathways in this study, as well as the involved DE genes.

## Abbreviations

KM: *K. marxianus* strain before evolution
KM-100d: *K. marxianus* population after 100-day evolution
NAD: nicotinamide adenine dinucleotide
NADP: nicotinamide adenine dinucleotide phosphate
GO: gene ontology
CoA: coenzyme A
TCA cycle: tricarboxylic acid cycle
ROS: reactive oxygen species
ER: endoplasmic reticulum
QC: quality control
DE: differentially expressed
RT-qPCR: real-time reverse transcription PCR
