# Supplementary material for: Kluyveromyces marxianus developing ethanol tolerance during adaptive evolution with significant improvements of multiple pathways
Source: Biotechnol Biofuels. 2019 Mar 22;12:63. doi: 10.1186/s13068-019-1393-z (PMC6429784; doi:10.1186/s13068-019-1393-z)
Supplement: Supplementary file 1 — Additional file 1: Fig. S1. The growth rate of KM and KM-100d at different ethanol concentrations. Fig. S2. Flow cytometer analysis for DNA content variation during K. marxianus evolution. Fig. S3. Heat map for log2ratio values with clustered genes and groups. Fig. S4. GO enrichment analysis for KM and KM-100d exposed in low and high ethanol. Fig. S5. Secretory pathway and cell wall biogenesis in K. marxianus pre- and post-evolution. Table S1. Primers for RT-qPCR analysis. [file 13068_2019_1393_MOESM1_ESM.docx]

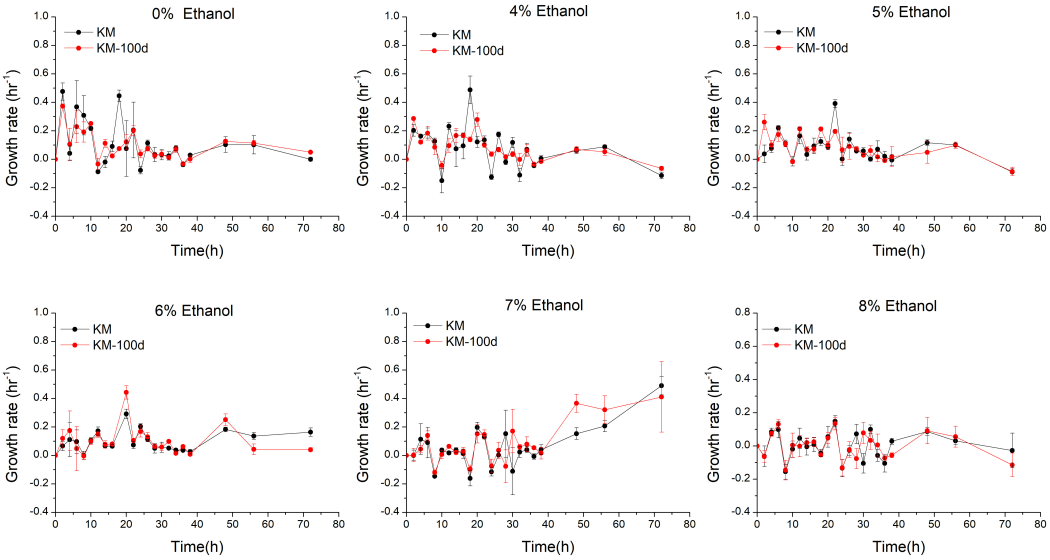


**Figure S1 Growth rate of KM and KM-100d at different ethanol concentrations.** In this figure, KM and KM-100d were both carried out in biological triplicate, and the specific growth rate for every replicate at each time point was calculated, and represented by the average value and mean variance. The red curve is for KM-100d, and the black one is for KM.


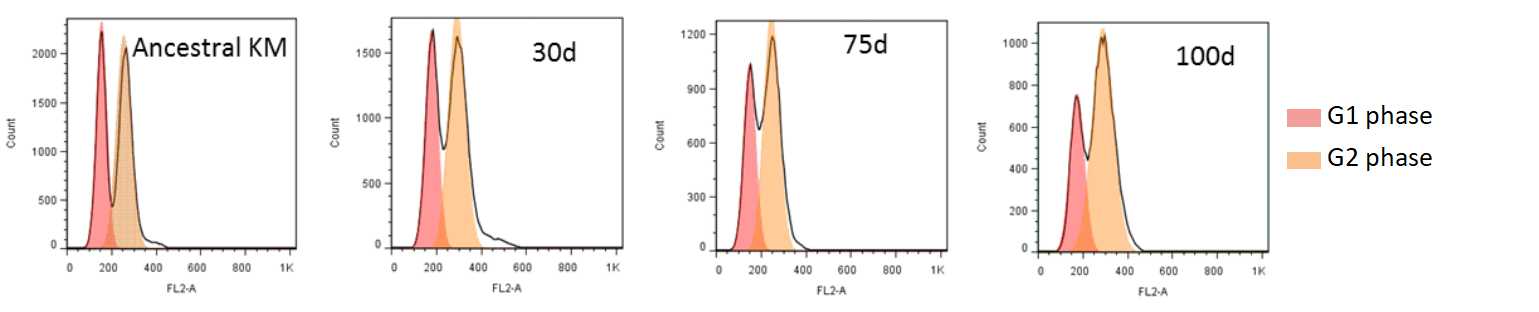


**Figure S2 Flow cytometer analysis for DNA content variation during *K. marxianus* evolution**. In this figure, the first left subfigure represents KM before evolution, and the subsequent subfigures display *K. marxianus* evolution population at 30 day, 75 day, and 100 day, respectively. It shows that, during the evolution course, DNA content of *K. marxianus* population has little alteration, i.e. there was no ploidy change during *K. marxianus’* adaptive evolution.


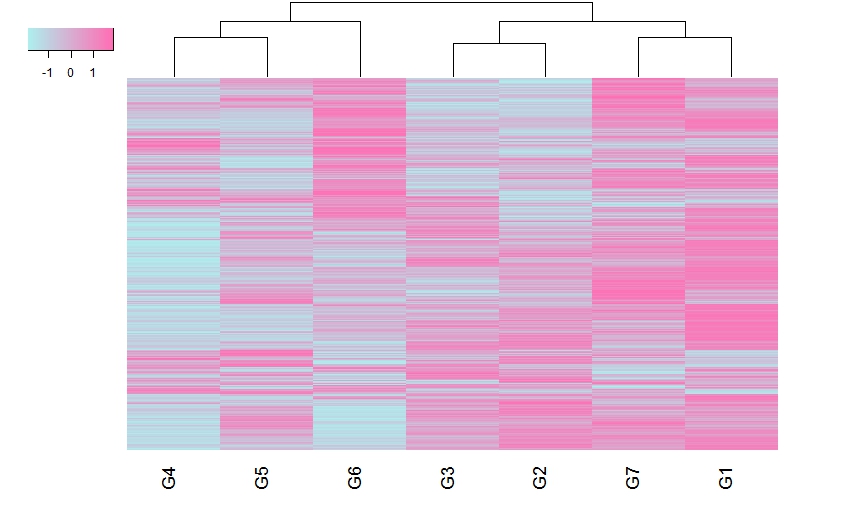


**Figure S3 Heat map for log_2_ratio values with clustered genes and groups**. In this figure, genes were denoted in rows, and groups were denoted in columns. Genes and groups were all reordered by heatmap.2 in R package to ensure similar patterns being next to each other. Group 1 is most close to Group 7. [Rose-red](https://fanyi.so.com/?src=onebox#%20rose-red) and sky blue refer to gene’s up- and down-regulation in a group, respectively. The group numbers are in line with those definitions in Fig. 2.


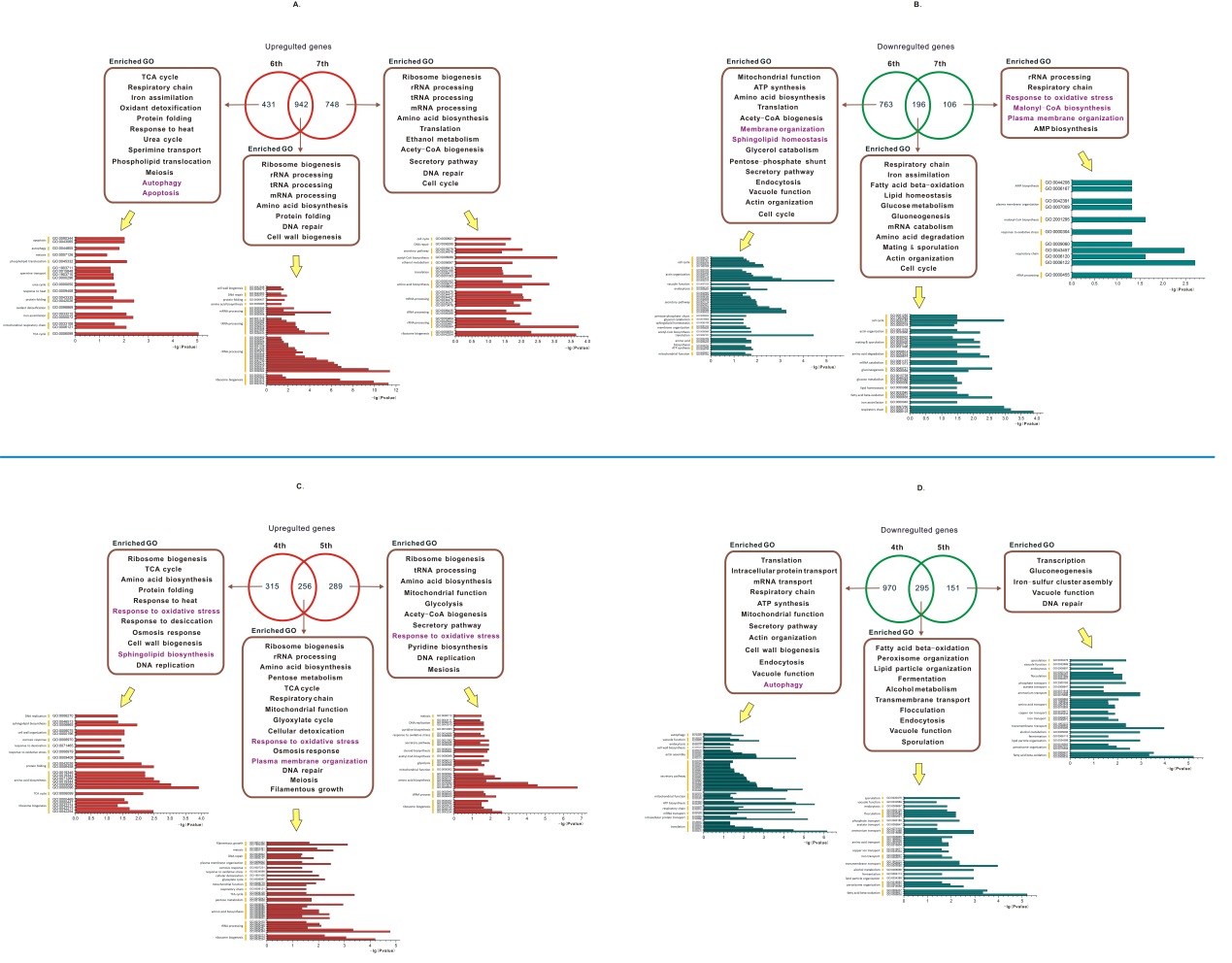


**Figure S4 GO enrichment analysis for KM and KM-100d exposed in low and high ethanol**. **(A)** GO enrichment of up-regulated genes in Groups ⑥ and ⑦. **(B)** GO enrichment of down-regulated genes in Groups ⑥ and ⑦. **(C)** GO enrichment of up-regulated genes in Groups ④ and ⑤. **(D)** GO enrichment of down-regulated genes in Groups ④ and ⑤. In each subfigure, the Venn diagram indicates the common and special genes for the two involved groups. Enriched GO pathways are listed in rectangles, and the nearby arrows point to the detailed enrichment information for the related GO terms.


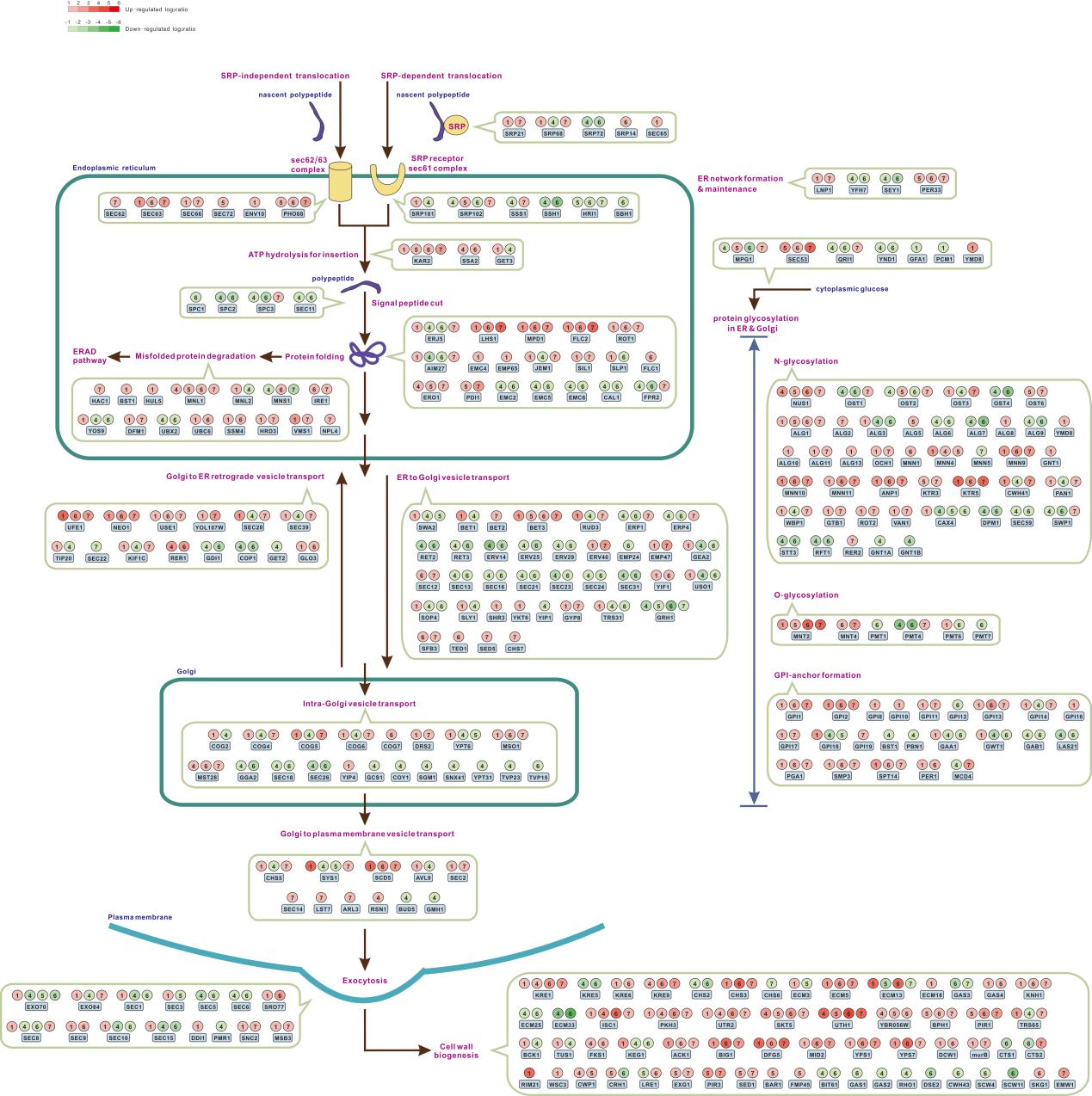


**Figure S5 Secretory pathway and cell wall biogenesis in *K. marxianus* pre- and post-evolution.** The major successive processes includes: nascent peptide are translocated into endoplasmic reticulum (ER) membrane through SRP (Signal Recognition Particle) dependent or independent ways, then signal peptide is cut, and protein is folded in ER. The properly folded protein goes on a vesicle-assisted travel, transported from ER to Golgi apparatus, from *cis*- to *trans*-Golgi, from *trans*-Golgi to cell membrane. When vesicle arrives at cell membrane, exocytosis takes place, vesicle lipids fuse into plasma membrane, and protein either stays on plasma membrane or to be released outside cell. During the secretory pathway, proteins are glycosylated in ER and Golgi, including N-glycosylation, O-glycosylation, and GPI anchor biogenesis, illustrated in the right part of the figure. The group numbers and colours are in line with those in Fig. 3.

**Table S1 Primers for RT-qPCR analysis**

| Gene | Primer |
| --- | --- |
| *SLN1* | TAACCAAAATGCTGGCGGGT |
|  | TGACATGCGCACAGATCCAT |
| *SSK1* | CAAGGTTGCAAAAGACGGGG |
|  | CAAGCCCAGAGAGTTTCGGT |
| *SSK2* | TCATCCGCTAACTCTTCCGC |
|  | AAGGGCTCCCGAGAGTTTTG |
| *TRR1* | GTCCCAGGCTCCTCTTTGAC |
|  | AAAGCGGCCATACAACCAGA |
| *GPX2* | GGGGCTTGAGATTCTTGCCT |
|  | GGCGTCACCTCCATTGACTT |
| *PEX6* | GGCTTGCGATGTTTGAGTGG |
|  | TAGAGGCGTTAAGCCAGCAG |
| *SKN7* | CTACCTCGCGGTTTCCATGT |
|  | GTCACGACTTCCACTGAGCA |
| *CNS1* | ATCGCAGTTGGTCTTCCCAG |
|  | AGGTTGAGCGAACCACTCTG |
| *PSD2* | TGTCGTGGATGGAACCCAAG |
|  | AGCTGGTCAATGTGTCGGAG |
| *CHO2* | GGCTATCGTACAACCTCGGG |
|  | CGAAGCGAGAGAGAGCTGAG |
| *LCB5* | GTGGTTGCAAAGTCGTCGTC |
|  | CCGGAAGCACAAGCAATGAC |
| *ERG27* | CCGCCACTTTCAGAGATGGT |
|  | ATAGTGCCGCTCTAGTTGGC |
| *PPT2* | CCAGCTCAAACCAAACGCAA |
|  | GTTCAATCTTGGCGATGCCC |
| *FAD2* | CGGTTTCATCGGTCCTCACA |
|  | GTAACGGTAGTGTTCGCCCA |
| *TAZ1* | TTGCAGTTGCAGCCTCCATA |
|  | ACCTGCACTTGATTCGGGAG |
